# Direct patterning of silver particles on porous silicon by inkjet printing of a silver salt via *in*-*situ* reduction

**DOI:** 10.1186/1556-276X-7-502

**Published:** 2012-09-06

**Authors:** Alessandro Chiolerio, Alessandro Virga, Paolo Pandolfi, Paola Martino, Paola Rivolo, Francesco Geobaldo, Fabrizio Giorgis

**Affiliations:** 1 Istituto Italiano di Tecnologia, Center for Space Human Robotics, Corso Trento 21, Torino, 10129, Italy; 2 Applied Science and Technology Department, Politecnico di Torino, Corso Duca degli Abruzzi 24, Torino, 10129, Italy; 3 Politronica Inkjet Printing S.r.L., Corso Castelfidardo 30/A, Torino, 10129, Italy

**Keywords:** Inkjet printing technology, Ag nanoparticles, Porous silicon, SERS

## Abstract

We have developed a method for obtaining a direct pattern of silver nanoparticles (NPs) on porous silicon (p-Si) by means of inkjet printing (IjP) of a silver salt. Silver NPs were obtained by p-Si mediated *in*-*situ* reduction of Ag^+^ cations using solutions based on AgNO_3_ which were directly printed on p-Si according to specific geometries and process parameters. The main difference with respect to existing literature is that normally, inkjet printing is applied to silver (metal) NP suspensions, while in our experiment the NPs are formed after jetting the solution on the reactive substrate. We performed both optical and scanning electron microscopes on the NPs traces, correlating the morphology features with the IjP parameters, giving an insight on the synthesis kinetics. The patterned NPs show good performances as SERS substrates.

## Background

Inkjet printing (IjP) technology has gained a great interest for the information technology compartment due to its high throughput and low costs [1]. Basically, it consists of a microelectromechanical system or a capillary where a piezoelectric actuator is able to produce a controlled droplet ejection. A servo-assisted head positioning makes it possible to additively draw complex structures and geometries in a way pretty similar to pattern printing by commercial printers. As a matter of fact, the fabrication of quite high-resolution patterns either on flexible substrates or on rigid ones, by means of this simple additive process, is highly attractive. Recent works report IjP as a new promising manufacturing technique for the fabrication of many different devices [2]. In the recent past, some electronic applications of IjP based on Ag NPs have been explored [3,4].

In the framework of useful applications, silver NPs synthesized directly on p-Si showed a good behavior as surface enhanced Raman scattering (SERS) substrates [5], which represent a high-sensitive label-free detection method in materials science, biophysics, medical diagnostics, and molecular biology [6]. In this specific application, no sintering is necessary. The performances of these types of substrates, dictated by their plasmonic response, depend on the morphology of the synthesized NPs that can be adjusted, controlling the concentration of silver nitrate, the process temperature, and the solvent typology impregnating the p-Si surface.

Herein is reported a study related to Ag NPs obtained by merging IjP technology with the redox reaction of AgNO_3_ solutions with p-Si surfaces. The morphology of the Ag NPs is correlated to the synthesis parameters. Such process opens new possibilities of simple fabrication concerning with metal-dielectric nanostructures aimed to sensing applications based on amplified Raman spectroscopy.

## Methods

### Samples synthesis

Porous silicon substrates were obtained by electrochemical etching of boron-doped silicon wafer (34 m(Ω)/cm) in HF solution (20:20:60 HF:H_2_O:CH_3_CH_2_OH) with a current density of 80 mA/cm^2^. We have obtained three solutions of AgNO_3_ having a concentration of 10^−2^ M using water (W), ethylene glycol (EG), and a solution composed of 50% water and 50% ethylene glycol (W:EG 1:1).

The so-prepared ink was printed by means of a piezoelectric Jetlab® 4 printer from MicroFab Technologies Inc. (Plano, TX, USA) equipped with a 60-μm nozzle diameter MJ-AT-01 dispenser. The print head was heated at 60°C, and the substrate was kept at room temperature during the deposition process to improve the quality of the deposit. The waveform used to expel a single droplet was a 35V pulse lasting 18 μs, followed by a −13V pulse lasting 36 μs as echo dwell with the rise, fall, and final rise time of 25, 5, and 2 μs, respectively. The step size was set to 100 μm, and 6 layers were printed on top of each other.

Concerning the drop on position (DOP) experiment, we used as ink a solution composed of 50% water and 50% ethanol with a AgNO_3_ concentration of 10^−2^ M; for the IjP parameters, we applied a waveform consisting of a 22V pulse lasting 20 μs, followed by a −15V pulse lasting 40 μs as echo dwell with the rise, fall, and final rise time of 5, 5, and 5 μs, respectively. During this process, the printing head is placed in a specific position of the plane and delivers a certain number of droplets in the same place, ejecting them at a specific frequency ranging within 250 Hz to 3 kHz.

### Sample characterizations

Scanning electron microscopy (SEM) images of Ag NPs on p-Si samples were obtained as secondary and backscattered electron images with 5- to 10-keV electrons using a Zeiss SUPRA 40 (Zeiss SMT, Germany) field emission electron microscope. Specular reflection spectra were obtained using an Agilent Cary 5000 (Agilent California, USA) UV-visible-NIR spectrophotometer equipped with a 12.5° reflectance unit, in the range of 200 to 2000 nm.

Raman spectra were obtained by means of a Renishaw inVia Reflex (Renishaw PLC, United Kingdom) micro-Raman spectrophotometer equipped with a cooled charge-coupled device camera. Samples were excited with an Ar-Kr laser source (514.5 nm), providing a photon flux lower than 60 W/cm^2^. The spectral resolution and integration time were 3 cm^−1^ and 10 s, respectively.

## Results and discussion

### Direct patterning of stripes

The synthesis of Ag NPs is based on the impregnation of p-Si in AgNO_3_ solutions through a redox process involving Ag^+^ cations with the hydride-covered surface [5,7,8], where the Ag reduction on the p-Si surface can be explained taking into account the following reaction for aqueous solutions:

$$\begin{array}{l} {\text{SiH}}_{\text{x}\left(\text{surf}\right)}+{\text{2H}}_{2}\text{O}+\left(4+\text{x}\right){\text{AgNO}}_{3\;}\\ \;\to \;{\text{SiO}}_{2\left(\text{surf}\right)}+\left(4+\text{x}\right){\text{HNO}}_{3}+\left(4+\text{x}\right)\text{Ag} \end{array}$$

**Figure 1:**
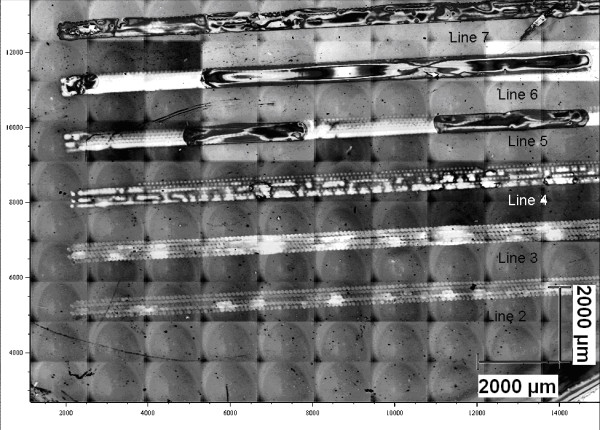
**Optical mosaic image of the IjP lines.** The contrast was increased by histogram equalization, helping to resolve the single droplet array in each line.

Concerning the W-based solutions, we obtained traces of Ag NPs, applying a double/multiple pass pattern since single IjP pass did not produce any NP synthesis. Both the pure H_2_O and the H_2_O:EG 1:1 solution resulted in a poor drop coalescence, where the occurrence of inter-drop necks increases with the pass numbers. The pure EG solution resulted in droplet coalescence and a complete NP stack formation, as determined by field emission scanning electron microscopy (FESEM). The pattern realized on p-Si consisted of strips, 15 mm in length and 500 μm in width (Figure 1).

**Figure 2:**
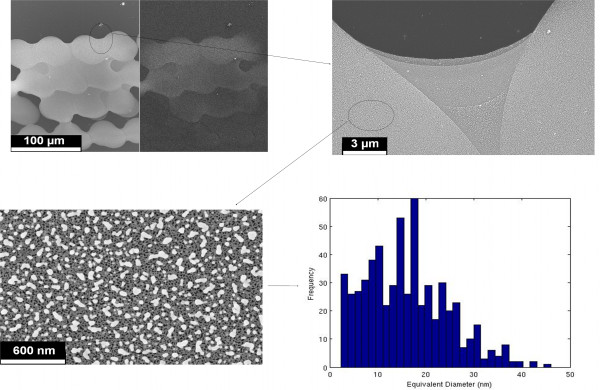
**Examples of SEM image analysis (performed on line 2).** Top left panel: magnified frame showing the droplet coalescence. Top right: selected area for the NP numerical analysis. Bottom left: high magnification micrograph depicting Ag NPs. Bottom right: NP diameter distributions.

By means of SEM analysis (see Figure 2 as example), we selected specific areas for the application of a numerical computation routine to be able to compute the NP size distribution [5]; obtained results are shown in Table 1.

**Table 1:** IjP stripe sample features

**Line number**	**Solvent carrier**	**Molarity (mol/l)**	**Viscosity (cP)**	**Number of passes**	**Average NP diameter (nm)**
1	W	10^−2^	1.5	1	N/A
2	W	10^−2^	1.5	2	40
3	W:EG 1:1	10^−2^	4	1	52
4	W:EG 1:1	10^−2^	4	1	17
5	W:EG 1:1	10^−2^	4	2	16
6	EG	10^−2^	18	1	16
7	EG	10^−2^	18	2	N/A

N/A, not available.

### DOP experiment

**Figure 3:**
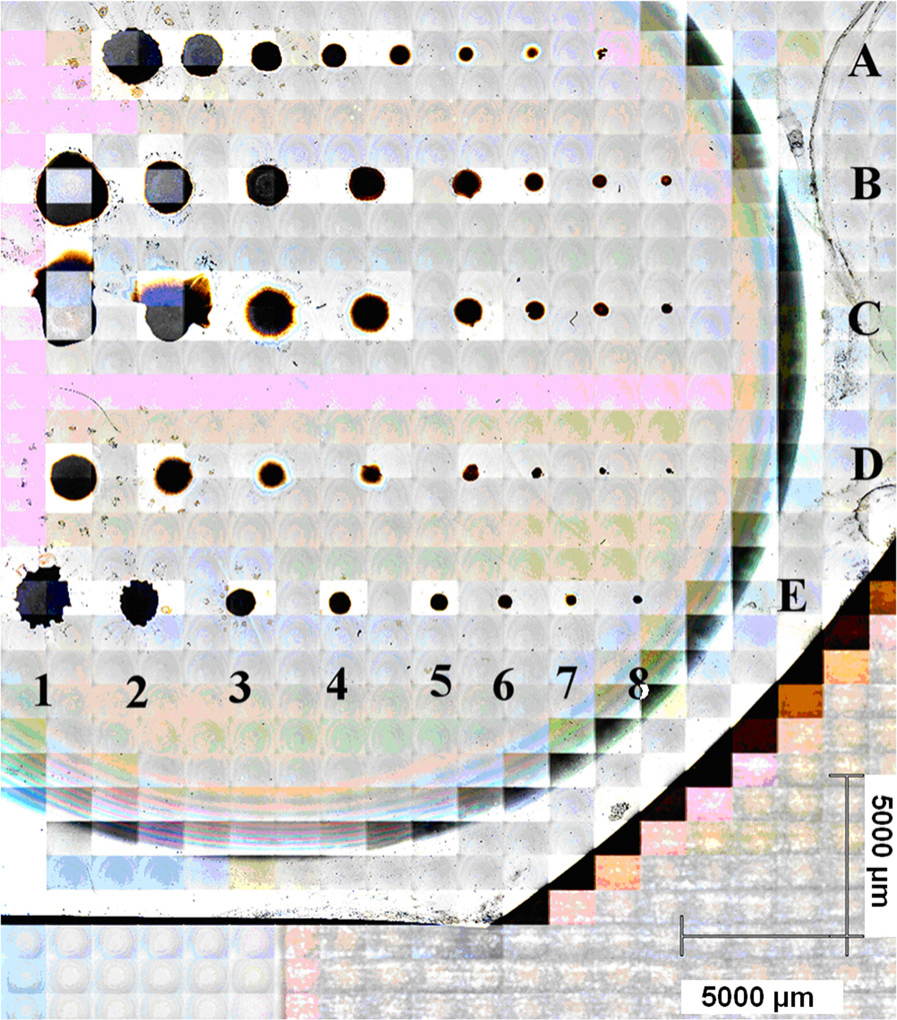
Optical mosaic image of the IjP DOP spots on p-Si, labeled in rows and columns.

A second set of experiments consisted in the DOP jetting of a controlled amount of reagents was performed in order to understand how the reaction kinetics may influence the NP size distribution and the related morphology. Actually, using the DOP dispensing technique, we could place a controlled amount of solution on a single spot, thus looking for the optimal liquid amount and reaction time, tuning the number of droplets jetted on a selected position and the ejection frequency. In Figure 3, we present some sample patterns, while in Table 2 we present the experimental conditions of IjP ejection frequency and number of droplets delivered for single position within the electrochemically etched silicon wafer, beside the correlated features concerning with the NP morphology as determined by FESEM. In particular, in order to compare different samples using a quantitative parameter, we modified a numerical algorithm previously used to determine the degree of percolation of NP populations [9,10] by computing a 2-D percolation factor (PF). We define PF as the ratio between the size of the major axis of the ellipse equivalent to the biggest particle recognized in the FESEM image and the mean frame size. The value of this factor tends to zero when the dispersion of isolated particles is homogeneous and is larger than 1 in the case of an infinite cluster found in the frame (i.e., an aggregate whose size is bigger than the frame size and whose coalesced particles cross all the FESEM image); it means that percolation is reached. This was done for a magnification of 25 kX, covering an area of 14.6 × 9.1 μm^2^. Besides such a parameter, the Ag filling fraction (FF) was calculated, as the percentage of Si surface covered by Ag particles, based on the SEM images.

**Table 2:** DOP sample IjP-ejection parameters and correlated features numerically determined from FESEM images

**Row**		**Line**							
		**1**	**2**	**3**	**4**	**5**	**6**	**7**	**8**
		**Drop number (*n***_**d**_**); Piezo actuation frequency (*f*, kHz)**							
		**2D-percolation factor (PF) and filling fraction (FF)**							
A	Drop number; (ND) frequency (F) (kHz)	5; 0.5	10; 0.5	20; 0.5	50; 0.5	100; 0.5	200; 0.5	500; 0.5	1,000; 0.5
	2-D percolation factor (PF)	0.3122	0.3814	0.0588	0.4035	0.41	0.3457	1.1612	1.1878
	Filling fraction (FF)	0.34	0.52	0.25	0.33	0.6174	0.32	0.49	0.51
B	Drop number; (ND) frequency (F) (kHz)	5; 1	10; 1	20; 1	50; 1	100; 1	200; 1	500; 1	1,000; 1
	2-D percolation	0.1523	0.2659	0.6141	1.3367	0.5059	1.2882	0.5416	1.1406
	Filling fraction (FF)	0.36	0.45	0.51	0.53	0.37	0.59	0.43	0.53
C	Drop number; (ND) frequency (F) (kHz)	5; 2	10; 2	20; 2	50; 2	100; 2	200; 2	500; 2	1,000; 2
	2-D percolation	0.9201	0.1943	0.9660	1.3438	0.8753	1.3546	1.9004	1.3843
	Filling fraction (FF)	0.5	0.4	0.52	0.61	0.44	0.7	0.63	0.62
D	Drop number; frequency (kHz)	5; 3	10; 3	20; 3	50; 3	100; 3	200; 3	500; 3	1,000; 3
	2-D percolation	0.4379	1.3328	0.6294	0.4142	0.8473	1.3184	1.1708	0.8992
	Filling fraction (FF)	0.43	0.56	0.51	0.5	0.56	0.55	0.48	0.58
E	Drop num; (ND) frequency (F) (kHz)	5; 0.25	10; 0.25	20; 0.25	50; 0.25	100; 0.25	200; 0.25	500; 0.25	1,000; 0.25
	2-D percolation	N/A	0.3670	0.3935	0.5767	0.8103	0.6106	1.1558	1.4360
	Filling fraction (FF)	N/A	0.48	0.37	0.37	0.29	0.38	0.39	0.52

**Figure 4:**
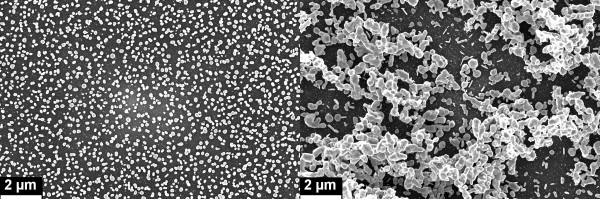
**FESEM images showing the Ag NP morphology on p-Si in different synthesis conditions.** IjP at 500 Hz, 20 droplets (**a**) and IjP at 1 kHz, 50 droplets (**b**).

In Figure 4a, we present a typical situation, consistent with a random distribution of Ag NPs that are well dispersed and are not in contact each other (20 droplets ejected at 500 Hz). In Figure 4b, the population is consistent with a coalescent geometry where multiple NPs form wide aggregates across the sample (50 droplets ejected at 1 kHz), sometimes yielding three-dimensional structures protruding from the sample surface.

FFs and PFs show a common trend versus the droplet number at several ejection frequencies. Actually, in order to quantify the correlation between PF and FF, we computed a 2-D cross-correlation factor (for a reference see [11]), obtaining a remarkable value of 0.7317, resulting from the numerical analysis of 40 different samples.

**Figure 5:**
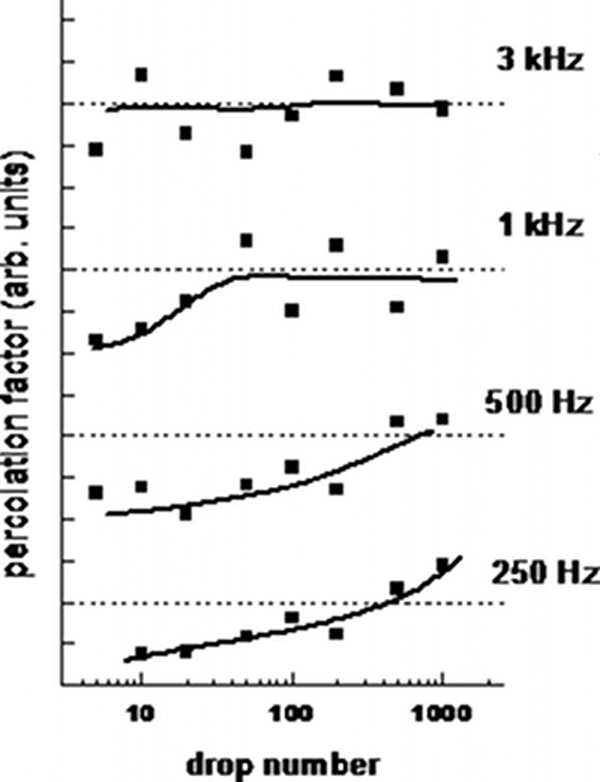
**2-D percolation factor for samples obtained with several droplet numbers and ejection frequencies.** The data corresponding to different frequencies are vertically shifted for the sake of clarity; the solid lines are guides for the eye, and the dashed lines represent the threshold of complete percolation.

Figure 5 shows the comparison of the percolation factor for most of the analyzed samples. As is straightforward to observe, for low ejection frequency (i.e., 250 Hz), there is a monotonical change of the nanostructure morphology which means a good control of the Si coverage by Ag NPs. By increasing the ejection frequency, after a certain threshold value concerning with the droplet number, PF oscillates around an average value. Such a threshold disappears for the highest frequencies (2 to 3 kHz), yielding a simple PF oscillation independently on the Ag amount. This does not mean that the nanostructures are no more evolving by increasing the droplet number since PF is a figure of merit dealing with a planar projection of the Ag NPs which tend to cluster also along the axis perpendicular to the p-Si substrate as clearly shown in Figure 4b.

### SERS measurements

In order to demonstrate the potential application of the ink-jet printed Ag NPs as active SERS substrates, we analyzed the optical response of a selected sample obtained using the same IjP parameters used for the above-mentioned stripes and an AgNO_3_ solution in pure water with a concentration of 10^−1^ M for the ink.

**Figure 6:**
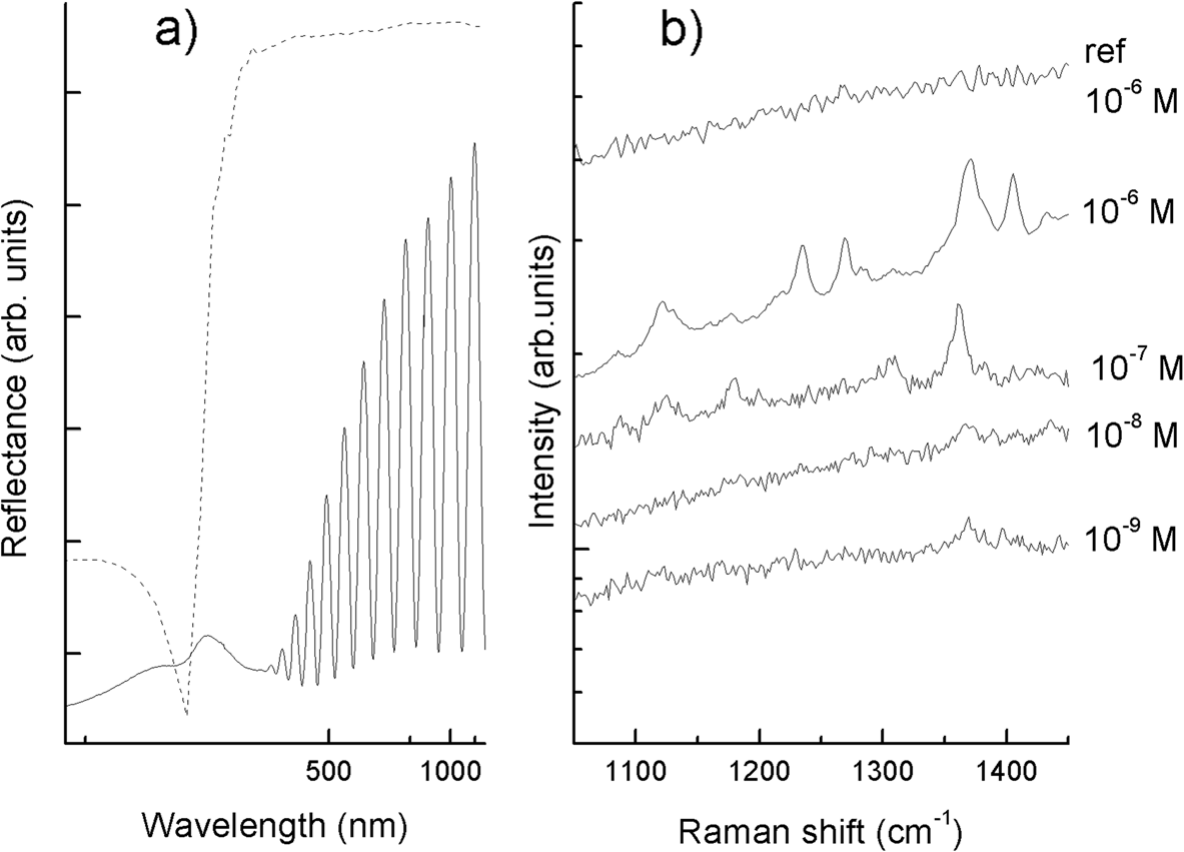
**Experimental reflectance spectrum and SERS spectra.** (**a**) Experimental reflectance spectrum of patterned Ag NPs (continuous line) compared to a calculation concerning with a homogeneous Ag thin film (dashed line). (**b**) SERS spectra of Cy-3-OCH_3_ dye at different concentration spotted on the same substrate. As a reference, the top graph shows the detected Raman spectrum of 10^−6^-M dye molecules on bare p-Si.

Figure 6a shows the specular reflectance spectrum characterized by a feature dip around 310 to 320 nm because of the Ag bulk plasmon (which can be clearly noticed in Ag thin films) and a wide additional dip (within the range of 360 to 520 nm) ascribed to either localized surface plasmons (LSP) coupled to individual particles or LSP due to interparticle short-range interaction. The influence of the interparticle plasmonic coupling, which is dictated by the nanometric distances among the particles and their ‘random’ local geometric features, makes a direct correlation between the particle morphology and the optical response difficult [5]. The interference fringes that are clearly observable in the reflectance spectrum at larger wavelengths are due to the p-Si layers supporting the Ag NPs.

It is widely proved that the main contribution to the SERS effect arises from an enhancement of the local electromagnetic field close to the metallic surface because of the excitation of LSPs [12]. Figure 6b shows the SERS spectra (excitation at 514.5 nm that is within the wavelength of the plasmonic dip shown in Figure 6a of a Cy3 dye adsorbed from aqueous solutions with several molar concentrations). The characteristic vibrational peak at 1,370 cm^−1^, ascribed to the −CH_3_ deformation mode (methyl groups are present as substituents on the Cy3 aromatic rings), is clearly detectable up to a concentration as low as 10^−9^ M, while as-anodized porous silicon substrates without Ag NPs do not show any Raman signal even for concentration of 10^−6^ M.

## Conclusions

Ag NPs on p-Si were obtained by means of IjP technology after *in-situ* reduction by means of chemical reaction between AgNO_3_ contained in the ink and p-Si substrate. Morphological characterizations and numerical calculation of some geometrical parameters were performed, showing that the pattern uniformity is strictly dependent on the ink viscosity (i.e., the type of solvent). In particular, for the same printing condition, higher ink viscosity facilitates the synthesis of particles and their coalescence. The DOP technique allowed the exploration of how the *in*-*situ* reduction leading to Ag NP formation is influenced by the number of droplets provided for each fixed position on the substrate and by the ejection frequency which modulate the reaction time and reactant flow rate. The correlation between such process parameters and NP population features was given in terms of 2-D percolation, numerically determined from FESEM images. It has been evidenced that for ejection frequency lower than 1 kHz, it is possible to control the nanostructure morphology by tuning the drop amount.

Finally, Ag NPs synthesized by IjP technique showed good performances as Raman enhancers. Thus, plasmonic patterns featuring a controlled nanostructure morphology could be exploited to produce arrayed elements active for SERS-based detection. Several potential applications in biomedicine can be foreseen such as microRNA SERS detection, where checking and quantifying different non-coding RNAs are important, and in a multiplexing label-free framework with the aim to actuate an early-cancer diagnosis [13].

## Abbreviations

DOP: drop on position; EG: ethylene glycol; FESEM: field emission scanning electron microscopy; FF: filling fraction; IjP: inkjet printing; LSP: localized surface plasmon; NP: nanoparticle; p-Si: porous silicon; PF: percolation factor; SERS: surface enhanced Raman scattering; W: water.

## Competing interests

The authors declare that they have no competing interests.

## Authors’ contributions

AC designed the experiment, performed the numerical analysis and correlations, and edited the paper. AV performed the ink-jet synthesis, Raman measurements, numerical analysis, and correlations. PP and PM carried out the ink-jet synthesis. PR and FrG designed the experiment. FaG also designed the experiment, executed the data analysis, and edited the paper. All authors read and approved the final manuscript.

## Authors’ informations

AC received his degree in Materials Engineering at Politecnico di Torino in 2005 and his Ph.D. in Electron Devices in 2009. He joined the χ-Lab Materials and Microsystems Laboratory research group, developing spintronic devices, MEMS and nanocomposite materials for electronic applications. AC is currently coordinating the technologies in support of the Smart Materials platform at Istituto Italiano di Tecnologia, with the aim of realizing a smart sensing skin. He is the co-author of more than 30 scientific papers, 3 national patents, 1 international patent, 7 book chapters and editor of 1 book. AV received his degree in Chemical Engineering at Politecnico di Torino in 2005 and his Ph.D. in Physics in 2011. The main field of interest is the production of silver and gold nanostructures, prepared through either nanosphere lithography or deposition, for SERS (surface enhanced Raman scattering) substrates. PP is an R&D engineer at Politronica S.r.L., a spin-off company of Politecnico di Torino (Italy), where he is the responsible of the Print Lab. In 2008, he obtained his masters degree in Materials Engineering. From 2008 to 2010, he worked at Politecnico di Torino as a fellow researcher in the Physics and Materials Science Department dealing with the deposition of thin films by means of vacuum techniques. Currently, his research activities are focused on printing technologies such as inkjet, R2R and pad printing of materials for electronic applications. He is a co-author of several scientific papers published on international journals. PM obtained her degree in Chemistry in 2001 at the University of Turin and her Ph.D. in Chemical Science in 2006. As post-doc, she joined the Spintronic research group at the Department of Physics (Politecnico di Torino), studying and developing magnetic devices, tunnel magnetoresistance and magnetic tunnel junctions (MTJs). At present, she works at Politronica S.r.L., a spin-off company of Politecnico di Torino, where she is responsible for the chemical laboratory. Her current area of research concerns with the synthesis and characterization of conductive, magnetic and dielectric inks for inkjet printing of electronic devices. She is the author and co-author of several scientific papers published on international journals. PR is an assistant researcher at the Applied Science and Technology Department of Politecnico di Torino (Italy), where she operates in the Materials and Microsystems Laboratory, a laboratory of Politecnico di Torino. In 1999, she obtained her masters degree in Chemistry. In 2003, she took her Ph.D. degree in Materials Science and Technology. Her research activities are focused on surface chemical modification of several materials (also porous and nanostructured), even by means of plasma-assisted techniques, in order to obtain hybrid organic–inorganic materials to be applied as optical and electromechanical sensors mainly for biological diagnostics. FrG is an associate professor at the Applied Science and Technology Department of Politecnico di Torino. He obtained his masters degree in Industrial Chemistry in 1990 and his Ph.D. in Chemical Science in 1995. His current area of research includes the synthesis and characterization of materials with special attention devoted to porous silicon for several applications. He is the author and co-author of more than 110 scientific papers published on peer-reviewed international journals. FaG is an associate professor of Physics of Condensed Matter at the Applied Science and Technology Department of Politecnico di Torino where he is in charge as research delegate. He obtained his masters degree in Physics in 1990 and his Ph.D. in Solid State Physics in 1995. He is a senior researcher at the χ-Lab Materials and Microsystems Laboratory of Politecnico di Torino and at the Istituto Italiano di Tecnologia - Center for Space Human Robotics. His current area of research concerns with the synthesis and characterization of silicon-based thin films in amorphous, micro-crystalline and porous phase with applications in photonic and plasmonic nanostructures. He is the author and co-author of more than 140 scientific papers published on peer-reviewed international journals.
